# Adolescent- and Young Adult-Reported Outcomes and Use of Continuous Glucose Monitoring Features: A Report from the CITY Trial

**DOI:** 10.1155/2023/6906023

**Published:** 2023-09-15

**Authors:** Laurel H. Messer, Colleen Bauza, Kellee M. Miller, Mark A. Clements, Daniel J. DeSalvo, Jennifer Sherr, Ruth S. Weinstock, Korey Hood, Lori M. Laffel

**Affiliations:** ^1^Barbara Davis Center for Diabetes, University of Colorado Anschutz Medical Campus (now with Tandem Diabetes Care), Aurora, CO, USA; ^2^Jaeb Center for Health Research, Tampa, FL, USA; ^3^T1D Exchange, St. Petersburg, FL, USA; ^4^Children's Mercy Hospital, Kansas City, MO, USA; ^5^Baylor College of Medicine, Houston, TX, USA; ^6^Yale School of Medicine, New Haven, CT, USA; ^7^SUNY Upstate Medical University, Syracuse, NY, USA; ^8^Stanford University, Stanford, CA, USA; ^9^Joslin Diabetes Center, Harvard Medical School, Boston, MA, USA

## Abstract

**Objective:**

To evaluate patterns of continuous glucose monitor (CGM) use and perceptions of quality of life in adolescents/young adults with type 1 diabetes (T1D) after using CGM for up to 52 weeks in the CGM Intervention in Teens and Young (CITY) Adults randomized clinical trial (RCT). *Subjects and Methods*. Participants with T1D were initially randomized 1 : 1 to use of CGM or blood glucose meter (BGM) for 26 weeks. Following the RCT, participants in the BGM group initiated CGM (BGM–CGM cohort) and participants in the CGM group continued CGM (CGM–CGM cohort) for another 26 weeks. Problem Areas in Diabetes Survey-Pediatric Version (PAID-peds), Glucose Monitoring Satisfaction Survey (GMSS), Hypoglycemia Confidence Scale (HCS), Diabetes Technology Attitudes (DTA), Pittsburgh Sleep Quality Index (PSQI), Benefits of CGM, and Burdens of CGM were completed at baseline, 26 and 52 weeks.

**Results:**

In both cohorts, >70% of participants were wearing CGM > 5 days/week at 52 weeks; 5% discontinued CGM. The majority used the mobile app to receive glucose data. Adolescents (14 to <19 years) were more likely to use SHARE features than young adults (80% versus 41%). CGM–CGM participants had significantly higher scores on GMSS, DTA, and HCS at 52 weeks compared with baseline, and reported higher benefit and lower burden perceptions than at baseline. Similar results were observed for the BGM–CGM cohort.

**Conclusions:**

Improvements in self-reported measures were observed in adolescents and young adults using CGM. As CGM use is also associated with better glycemic control, utilizing CGM may contribute to improving both medical outcomes and emotional health.

## 1. Introduction

Across the lifespan, adolescence and young adulthood (ages 14–24 years) are characterized as the period challenged most with achieving optimal hemoglobin A1c (HbA1c) levels for people with type 1 diabetes (T1D), with less than 14% meeting the American Diabetes Association target of HbA1c < 7% (<53 mmol/mol) [1, 2]. Recently, the Continuous Glucose Monitors (CGM) Intervention in Teens and Young (CITY) Adults with T1D study by Laffel et al. [3] demonstrated the beneficial impact CGM can have in this age group, with CGM users achieving a 0.37% reduction in HbA1c compared with non-CGM users. The United States (US) T1D Exchange Registry data also indicate that CGM use is associated with lower mean HbA1c compared with no CGM use [1].

Despite the recognized glycemic improvement with CGM use, T1D Exchange data from 2017/2018 showed only 24% of adolescents (age 13–<18) and 22% of young adults (age 18–<26) used CGM [1]. Identified barriers to adolescents using diabetes technologies include cost and insurance-related issues, the perceived hassle of wearing the device, dislike of the device on the body, and feeling nervous that the device may not work [4]. There are likely other important factors to consider as well, as the adolescent/young adult period is uniquely marked by profound developmental, physiological, and social changes [5]. Considering the importance of CGM use as a tool to improve glycemia in this uniquely vulnerable population, it is imperative to further understand how adolescents/young adults use CGM in this period of life, as well as person-reported outcomes (PROs) that may elucidate common barriers and facilitators of using CGM. The CITY trial therefore sought to more thoroughly assess the adolescent/young adult experience with CGM and evaluate the CGM use patterns in adolescents/young adults after using CGM for up to 12 months.

## 2. Materials and Methods

This trial was conducted at 14 US endocrinology practices after approval by institutional review boards. Details of the study design have been previously published by Laffel et al. [3], and 12-month clinical outcomes were previously reported by Miller et al. [6]. The full protocol is available at https://public.jaeb.org/datasets and details are provided on the clinicaltrials.gov (NCT03263494). In brief, participants 14 to less than 25 years old with T1D for at least 1 year, who used either insulin pump or multiple daily injections and had not used CGM in the prior 3 months were randomized 1 : 1 to use of CGM (Dexcom G5, Dexcom, San Diego, CA) or to use a downloadable home blood glucose meter (BGM) without real-time, personal CGM for 26 weeks. Participants completing the 26-week randomized clinical trial (RCT) entered an extension phase and were followed for an additional 26 weeks for a total of 52 weeks of follow-up. In this extension phase, the BGM group initiated CGM use (referred to as BGM–CGM cohort) and the CGM group continued CGM use (CGM–CGM cohort); all participants used the Dexcom G6 CGM (Dexcom, Inc., San Diego, CA) in the extension phase.

## 3. Person-Reported Outcomes

PROs were measured using the following instruments.

### 3.1. Problem Areas in Diabetes Survey—Pediatric Version (PAID-Peds) [7]

PAID-peds measures diabetes-related emotional distress. The instrument includes 20 items rated on a 5-point Likert scale ranging from “not a problem” to “big problem.” The total score ranges from 0 to 100, with higher scores indicating greater diabetes-related emotional distress.

### 3.2. Glucose Monitoring Satisfaction Survey (GMSS) [8]

The GMSS includes 15 items rated on a 1–5 scale and generates a total score and four subscale scores including: openness, emotional burden, behavioral burden, and trust. Higher total scores indicate greater satisfaction in the current glucose monitoring modality.

### 3.3. Hypoglycemia Confidence Scale (HCS) [9]

The HCS has nine questions that assess the degree to which people with diabetes are confident regarding their ability to manage hypoglycemia. Each of the scale's nine items are rated on a 0–3 scale, from not at all confident to very confident. Higher scores indicate more confidence in ability to manage hypoglycemia.

### 3.4. Diabetes Technology Attitudes (DTA) [10]

The DTA contains five questions rated on a 5-point scale and measures attitudes toward diabetes technology. Higher scores indicate a more positive attitude toward diabetes technology.

### 3.5. Pittsburgh Sleep Quality Index (PSQI) [11]

The PSQI assesses sleep quality during the previous month and provides a global score ranging from 0 to 21 based on the seven component scores (each scored 0–3). Higher scores indicate worse sleep quality.

### 3.6. Benefits of CGM (BenCGM) and Burdens of CGM (BurCGM) [12]

The BenCGM and BurCGM surveys measure the user-perceived benefits and BurCGM use. Each instrument includes eight items rated on a 1–5 scale. Higher scores on each measure indicate that personal CGM use and remote monitoring of CGM data are more of a benefit or burden, respectively.

## 4. Statistical Analysis

Descriptive statistics were used to summarize all data. For the CGM–CGM cohort, CGM use and device settings were tabulated at the 6, 13, 26, 39, and 52-week visits. For the BGM–CGM cohort, CGM use and device settings were determined at the 39 and 52-week visits. PROs were reported at the RCT baseline, 26 and 52 week visits for both cohorts. A paired *t*-test or signed rank test, as appropriate, was used to evaluate the change in CGM use and prespecified PROs between baseline and 52 weeks and between 26 weeks and 52 weeks in the CGM–CGM cohort and between 26 weeks and 52 weeks in the BGM–CGM cohort.

Additional post hoc analyses were included to assess the association of sharing through the Dexcom mobile application among mobile application users with the following outcomes at 52 weeks: GMSS, BenCGM, BurCGM, and CGM hours. For each outcome, a multivariable linear regression model was fit with the continuous outcome at 52 weeks as the dependent variable and use of sharing through the mobile application and treatment group included as predictors.

Two-sided *P*-values were used and were adjusted for multiple comparisons to control for the false discovery rate using the adaptive Benjamin–Hochberg procedure [13]. All analyses were conducted on available cases only and included those who completed the 52-week visit. Analyses of CGM use were limited to those with nonmissing data on CGM use, and analysis of the use of CGM features were restricted to those with nonmissing and nonzero data on CGM use. RCT baseline refers to prerandomization measurements. Analyses were conducted with SAS software version 9.4 (SAS Institute Inc., Cary, NC).

## 5. Results

Of the 142 participants who completed the 26-week RCT phase, 140 completed the additional 26 weeks of follow-up. The age range of the 140 participants at entry into the extension phase was 14–25 years, 49% were female, 63% non-Hispanic white, and 58% had private insurance. Characteristics at baseline have been previously reported and characteristics at the start of the extension phase (26 weeks) for the two cohorts are shown in Table S1.

### 5.1. CGM Use for Both Cohorts

CGM–CGM participants were using the Dexcom G5 CGM for a median average of 6.6 days/week in the 28 days prior to the 26-week visit, and the G6 device 6.8 days/week by week 52 (*P*=0.60, Table 1). BGM–CGM participants were using the G6 CGM for a median average of 7.0 days/week in the 28 days prior to the 39 and 52-week visits (Table 1). In both cohorts, more than 71% of participants were wearing CGM > 5 days/week at 52 weeks, and 97% were using the CGM to make diabetes management decisions (in lieu of fingerstick blood glucose checking). At 52 weeks, the majority of participants had the low-glucose alert turned on 91% in the CGM–CGM group and 80% in the BGM–CGM group. Eleven percent of participants in each group used different low-alert settings during the day versus at night. Overall, the low-alert setting at 52 weeks ranged from 60 to 90 mg/dL (3.3–5.0 mmol/L) in both cohorts (CGM–CGM and BGM–CGM, median 70 mg/dL; IQR 70, 75 mg/dL (3.9 mmol/L; 3.9, 4.2 mmol/L)).

About 80% of participants in both cohorts had the high-glucose alerts activated at the 52-week visit (81% in the CGM–CGM group and 78% in the BGM–CGM group). A small portion of participants programed different high-alert settings during the day versus at night (11% in the CGM–CGM and 9% in the BGM–CGM group). The high-alert setting at 52 weeks ranged from 180 to 400 mg/dL (10.0–22.2 mmol/L) in the CGM–CGM group, median 250 mg/dL; IQR 205, 300 mg/dL) (13.9 mmol/L (11.4, 16.7 mmol/L)) and from 160 to 400 mg/dL (8.9–22.2 mmol/L) in the BGM–CGM group, median 300 mg/dL; IQR 250, 300 mg/dL (16.7 mmol/L; 13.9, 16.7 mmol/L).

### 5.2. Effect of Using the SHARE Application

By 52 weeks, 88% of the CGM–CGM group and 68% of the BGM–CGM group were using the Dexcom G6 Mobile App, the remainder accessed CGM data using a receiver provided through the study. Of the participants using the mobile application, over 60% chose to share their glucose values with another person using the Dexcom SHARE/FOLLOW applications (Table 1). There was no significant difference in CGM hours among participants who used the SHARE application compared with those who did not (*P*=0.34) (Table S2). No differences in GMSS, CGM benefits, or CGM burdens were noted between SHARE users and nonusers.

The 30 participants who were not using the mobile application at 52 weeks reported the following reasons: does not own a smartphone (20%), issues with WIFI/data plan (3%), smartphone does not support mobile application (33%), preferred the receiver (20%), battery life concerns on the phone (3%), trouble getting mobile application started/synced (10%), and storage/connectivity issues on the phone (10%). The 34 participants reporting not using the SHARE feature at 52 weeks cited the following reasons: not wanting someone else to see glucose values/receive alerts (56%), never set it up (12%), significant other/friend/family member does not want to see glucose values/see alerts (9%), lack of phone capability (9%), issues with technology/battery life (6%), no other person was responsible for diabetes care (6%), and lack of family technology knowledge (3%).

### 5.3. CGM Use by Adolescents (14 to <19 Years at Screening) and Young Adults (19 to <25 Years at Screening)

Over 75% of participants in both age groups (14 to <19 years, 19 to <25 years) were using the mobile application at 52 weeks (Table 2). Adolescent participants 14 to <19 years appeared more likely to use the SHARE feature (80%) compared with the young adult participants 19 to <25 years old (41%, post hoc comparison not calculated). Over 80% of adolescents were sharing their glucose values with only their parent compared with 36% of young adults. Further, a higher percentage of adolescent participants (65%) felt that it was helpful to have others following their glucose values compared with the young adult participants (17%).

### 5.4. Person-Reported Outcomes in CGM–CGM Cohort

CGM–CGM participants reported significantly higher glucose monitoring satisfaction, diabetes technology attitudes, and hypoglycemia confidence at 52 weeks compared with the RCT baseline (*P* ≤ 0.01 for all; Table 3). There was a trend toward a reduction in diabetes burden (PAID) from RCT baseline to 52 weeks (*P*=0.05). Additionally, the CGM–CGM group perceived CGM use to be more beneficial and less burdensome at 52 weeks than at baseline (*P* < 0.001). No difference in sleep quality was observed from the RCT baseline to 52 weeks. For all PROs, no differences were observed from 26 weeks to 52 weeks.

### 5.5. Person-Reported Outcomes in BGM–CGM Cohort

BGM–CGM participants reported significantly higher glucose monitoring satisfaction and diabetes technology attitudes at the 52 week time point, after 26 weeks of using CGM, compared with extension phase baseline prior to CGM use (at 26 weeks) (*P* < 0.01 for all; Table 4). There was no change in diabetes burden (PAID) from 26 to 52 weeks (*P*=0.08). Additionally, the BGM–CGM group perceived CGM and SHARE use to be more beneficial and less burdensome at 52 weeks than at 26 weeks (observational extension baseline). No differences were observed for hypoglycemia confidence or sleep quality from the observational extension baseline of 26 weeks to 52 weeks.

## 6. Discussion

To our knowledge, this is the most comprehensive report of CGM usage patterns and PROs among adolescents and young adults with T1D using CGM over a 52-week period. Regardless of duration of device wear, 26 weeks in the BGM–CGM group and 52 weeks in the CGM–CGM group, more than three quarters of each group persisted with CGM wear >5 days/week by the end of the study. More importantly, the participants were not just wearing the devices, the majority were using high- and low- glucose alerts, suggesting they were using the real-time data generated to enact changes in the glycemic management. Many integrated the use of CGM through the mobile app and, especially in adolescents to <19 years, SHARE capabilities were frequently utilized. Participants reported more hypoglycemia confidence and across-the-board technology satisfaction.

In 2008, the landmark JDRF randomized control trial demonstrated the clinical benefits of CGM use. Yet, it became evident that this was not true across all age groups studied. Indeed, that trial demonstrated that adolescents and young adults wore the CGM less than other age groups, averaging around 2.5 days/week for 26 weeks [14]. By contrast, the CITY study shows high consistency of CGM use with median CGM wear noted to be 6.8 days/week in the CGM–CGM cohort and 7.0 days/week in the BGM–CGM group. Further, the 2008 study reported no improvement in PROs among children or adults from baseline to 26 weeks (including diabetes distress or CGM satisfaction [15]) whereas, this study showed improvement in many measured PRO domains. This is likely attributable to the advances in the CGM technology. Technology acceptance theory indicates that a person is likely to use a technology if they perceive it as useful and also easy to use [16, 17]. Due to the features that are embedded in modern CGMs, it is likely that the participants in this trial found more benefit and ease with CGM than the original 2008 trial.

The CGM systems used in the CITY trial were the Dexcom G5 and G6, which offer significantly improved features relative to prior models. First, the CGM is approved to replace blood glucose monitoring for diabetes management decisions (e.g., guiding insulin doses to correct high-glucose levels), and 97% of participants in this study took advantage of this indication. Checking blood glucose levels routinely is a struggle for adolescents [18]. The majority of participants downloaded the mobile application so they could receive glucose values on their mobile phone instead of relying on a standalone receiver. This may be an important facilitator for adolescents and young adults who are heavily influenced by the peer acceptance [19, 20], and may find it more socially acceptable to discretely look at CGM values on a cell phone as opposed to pulling out a separate medical device. Socioeconomic status may impact ability to have a smartphone able to connect to CGM devices, and this may impact CGM use in some persons with diabetes. Both low- and high-glucose alerts were utilized by the majority of participants throughout the trial. The median glucose value for the low alert was consistently at 70 mg/dL (3.9 mmol/L) at all timepoints, which is the commonly used threshold of level 1 hypoglycemia in diabetes [2, 21], and is often suggested as the low-alert threshold in the clinical practice. The median glucose value for the high-alert fluctuated between 240 and 300 mg/dL at various timepoints, with the BGM–CGM group using 300 mg/dL for 52 weeks. Alert fatigue is a common barrier to consistent diabetes device use [4, 10], and clinicians should employ shared decision-making strategies to set personalized alert thresholds in persons with diabetes. The parameters commonly used by participants in this study (e.g., low alert of ∼70 mg/dL and high alert of 240–300 mg/dL) may provide initial tolerable limits to consider for adolescents and young adults who are new to CGM. It is possible that use of the high alert contributed to the improved glycemic control achieved in this study, either by helping adolescents and young adults recognize and treat hyperglycemia in a timely manner or could have aided with engagement in care to prevent episodes of the persistent hyperglycemia.

Improved usability features and high-wear time of the current CGMs may explain the improvement in psychosocial measures as well. Diabetes distress is a well-known phenomenon in diabetes and is associated with changes in the metabolic control [22, 23]. While the improvement in diabetes distress did not meet statistical significance, possibly due to multiple comparison adjustments, further research is warranted. Diabetes distress measures, to date, have not been sensitive to changes with the initiation of diabetes technology, which is why even the trend toward significance seen in this study is an encouraging finding. Hypoglycemia confidence improved significantly in the CGM–CGM group over 1 year but not in the BGM–CGM over 6 months. This may indicate that achieving an increase in hypoglycemia confidence takes a longer period of time [24, 25]. More advanced technologies including automated insulin delivery systems with ability to attenuate or suspend insulin delivery for down trending glucose may further improve hypoglycemia confidence.

This study occurred (2018) prior to the more recent trend for CGM to be initiated close to onset of T1D. As such, the study population represented a wide variety of participants, not necessarily “technology laggards” who were more hesitant to use technology [26]. Some of the participants had experience with older CGM devices, and still found positive benefit from using CGM in this trial. Measures of device satisfaction were across-the-board positive and improved throughout the 1 year for the CGM–CGM group and the 6 months for the CGM–CGM group, which may have been impacted by the change from G5 to the G6 model. The timing of this study (2018) was at a turning point for CGM technology with the wider availability of mobile phone viewing, remote monitoring, and replacement of blood glucose monitoring for insulin dosing. The technology attitudes scores improved from baseline in both groups and were higher than reported in the real-world use [4]. The BenCGM scores improved as well and were also very high (high-perceived benefit) across time, including the baseline assessment. At baseline, the BurCGM scores demonstrated low-perceived burden yet these measures still improved after wearing CGM. These results are encouraging and may indicate that perceptions of high benefit and low burden contribute to the ongoing wear of CGM. This is theoretically supported in both the health belief model [27, 28], for health behavior and the technology acceptance model [16, 17], both of which acknowledge benefit/usefulness and burden/ease of use contributing to a person's intention to perform a behavior or use a device. There were not enough discontinuers to meaningfully assess differences in perceptions of benefit and burden; yet future trials should continue to explore characteristics of the population who discontinue to those who persist with a particular focus on these topics.

Strengths of this study include the thorough exploration of CGM use characteristics and thoughtfully chosen psychosocial outcomes in a large cohort of adolescents and young adults with T1D. The data were collected for up to 12 months, providing longitudinal understanding of perceptions and durability of CGM use. Recognizing the rapidity with which technology evolves, the trial was designed to include the latest generation CGM available and thus by the end of the 6-month RCT all participants were provided the Dexcom G6. There are notable limitations as well. Because CGM use was very high by the majority of participants, it was not possible to determine if perceptions or system use attributes differed between those who wore CGM less frequently and those who wore it more continuously. We were not able to assess meaningful attributes of CGM discontinuers since <6% of the cohort discontinued use. Further, as this was a structured clinical study where CGM was provided free of charge to research participants, there are limitations to real-world implications in terms of access, affordability, and ability to use continuously. Real-world studies will provide further insight into the impact of insurance status and accessibility of CGM long term.

In conclusion, the CITY trial provided evidence of the successful integration of CGM use in the adolescent and young adult population to impact glycemia. While providers may place great importance on glycemia, it is also critical to understand how PROs can be impacted by the new therapies. The present analysis highlights that positive PRO changes accompanied by persistent CGM use can be achieved in this age group. Understanding the high use of the mobile app, sharing data with others, and alert settings may provide meaningful guidance to clinicians attempting to optimize use of CGM in young people with diabetes. Empiric knowledge that improvements in diabetes distress and hypoglycemia confidence are possible can be shared in the clinical practice to help others integrate CGM and potentially other advanced diabetes technologies. Indeed, this may help shape the future narrative in clinical care: CGM is accepted and effectively used in adolescents and young adults leading to meaningful improvements in the glycemic control and psychosocial outcomes. With technological advances continuing to evolve, including use of automated insulin delivery systems in the clinical care, demonstrating the persistence of CGM wear highlights that adolescents and young adults may stand to benefit from integration of more advanced therapies.

## Figures and Tables

**Table 1:** CGM use and device feature use.

	CGM–CGM						BGM–CGM	
	RCT phase			Extension phase		*P*-value 26 (dexcom G5) vs. 52 (dexcom G6) weeks ^*∗*^	Extension phase	
	6 Week	13 Week	26 Week	39 Week	52 Week		39 Week	52 Week
CGM use (%)								
*N*^†^	70	70	70	70	70		70	70
Median % of time (Q1, Q3)	86% (64%, 94%)	76% (41%, 92%)	79% (46%, 94%)	88% (64%, 95%)	86% (55%, 95%)	—	90% (75%, 96%)	91% (62%, 95%)
Median # days/week (Q1, Q3)	7.0 (6.0, 7.0)	6.6 (4.0, 7.0)	6.6 (4.3, 7.0)	6.5 (5.3, 7.0)	6.8 (4.8, 7.0)	0.60	7.0 (6.0, 7.0)	7.0 (5.3, 7.0)
*N* (%) wearing <5 days	12 (17%)	19 (27%)	20 (29%)	14 (20%)	20 (29%)		9 (13%)	16 (23%)
*N* (%) wearing ≥5 days	58 (83%)	51 (73%)	50 (71%)	56 (80%)	50 (71%)		61 (87%)	54 (77%)
Discontinuation of CGM (wearing 0%)	2 (3%)	3 (4%)	6 (9%)	5 (7%)	6 (9%)		2 (3%)	1 (1%)
Device feature use								
*N*^‡^	—	66	64	65	64		—	69
Mobile application	—	49 (74%)	52 (81%)	—	56 (88%)	—	—	47 (68%)
*N*^‡^	—	49	52	—	56		—	47
SHARE feature among mobile application users	—	28 (57%)	32 (62%)	—	35 (63%)	—	—	34 (72%)
Alarm use								
*N*^‡^	23	46	64	65	64		68	69
Low alert on	23 (100%)	44 (96%)	58 (91%)	60 (92%)	58 (91%)		53 (78%)	55 (80%)
High alert on	20 (87%)	40 (87%)	54 (84%)	53 (82%)	52 (81%)		52 (76%)	54 (78%)

^*∗*^*P*-values are from a signed-rank test. *P*-values are adjusted for multiple comparisons to control the false discovery rate (FDR). ^†^Only participants who completed and had data available at the 52-week visit are included in the tabulation of CGM use. ^‡^Only participants who had nonzero and nonmissing CGM use are included in the tabulation of feature use.

**Table 2:** Device feature use and application details at 52 Weeks stratified by adolescents and young adults.

	14 to <19 years	19 to <25 years
	52 Week	52 Week
Device feature use ^*∗*^		
*N*^†^	89	44
Mobile application	69 (78%)	34 (77%)
*N*^‡^	69	34
SHARE feature among mobile application users	55 (80%)	34 (41%)
Alarm use ^*∗*^		
*N*^†^	89	44
Low alert on	78 (88%)	35 (80%)^†^
High alert on	71 (80%)	35 (80%)
*N*^‡^	87	42
Uses different low alarm day vs. night	9 (10%)	5 (12%)
Uses different high alarm day vs. night	8 (9%)	5 (12%)
SHARE application details^‡^		
*N*^†^	55	14
Median # of people following CGM data using SHARE/FOLLOW (Q1, Q3)	1 (1, 2)	2 (1, 2)
People who following participant on SHARE/FOLLOW application	55	14
Parent only	44 (80%)	5 (36%)
Parent + partner/spouse/significant other	2 (4%)	3 (21%)
Parent + partner/spouse/significant other + roommate	0 (0%)	1 (7%)
Parent + sibling	0 (0%)	2 (14%)
Parent + roommate	1 (2%)	0 (0%)
Parent + aunt	3 (5%)	0 (0%)
Parent + school personnel/coach	1 (2%)	0 (0%)
Partner/spouse/significant other only	0 (0%)	3 (21%)
Sibling only	1 (2%)	0 (0%)
Grandmother + aunt	1 (2%)	0 (0%)
Friend only	2 (4%)	0 (0%)
Alerting Participant	55	14
Someone using SHARE/FOLLOW application alerted participant of low	49 (89%)	8 (57%)
Someone using SHARE/FOLLOW alerted participant of high	47 (85%)	5 (36%)
Miscellaneous	55	14
Helpful to have others following CGM data	45 (65%)	12 (17%)
Stopped sharing data through SHARE/FOLLOW	3 (5%)	0 (0%)
Median # of people who participant removed from SHARE/FOLLOW application (Q1, Q3)	1 (1, 1)	—

^*∗*^Only participants who completed and had data available at the 52-week visit, had nonzero and nonmissing CGM use are included in the feature use and alarm use tabulations. ^†^Only participants who had nonzero and nonmissing CGM use are included in the tabulation of feature use. ^‡^Only participants who completed and had data available at the 52-week visit, had nonzero, nonmissing CGM use, and were using the mobile application are included in the SHARE application details tabulations.

**Table 3:** Person-reported outcomes for the CGM–CGM group.

	CGM–CGM ^*∗*^											
		RCT baseline			26 Weeks (observational extension baseline)			52 Weeks			*P*-value RCT baseline vs. 52 weeks	*P*-value 26 vs. 52 weeks
	Description	*N*	Median (Q1, Q2)	Mean ± SD	*N*	Median (Q1, Q2)	Mean ± SD	*N*	Median (Q1, Q2)	Mean ± SD		
PAID^†^	Score ranges 0–100; higher score indicates more of a problem	69	21.0 (10.0, 39.0)	26.9 ± 2.7	69	19.0 (7.0, 40.0)	25.5 ± 2.8	69	17.0 (7.0, 35.0)	22.8 ± 2.5	0.05^‡^	0.23^‡^
Glucose monitoring satisfaction	Score ranges 1–5; higher score indicates more satisfaction	69	3.8 (3.3, 4.1)	3.7 ± 0.1	69	3.9 (3.5, 4.5)	4.0 ± 0.1	69	4.1 (3.7, 4.5)	4.0 ± 0.1	<0.001^§^	0.66^§^
Hypoglycemia confidence^†^	Score ranges 1–4; higher score indicates more confidence	69	3.7 (3.0, 4.0)	3.4 ± 0.1	69	3.7 (3.1, 4.0)	3.5 ± 0.1	69	3.8 (3.3, 4.0)	3.6 ± 0.1	<0.001^‡^	0.11^‡^
Diabetes technology attitudes	Score ranges from 6 to 30; higher score indicates a more positive attitude toward diabetes technology	69	24.0 (20.0, 25.0)	22.2 ± 0.4	69	25.0 (22.0, 25.0)	23.2 ± 0.4	69	25.0 (23.0, 25.0)	23.1 ± 0.5	0.01^‡^	0.94^‡^
Sleep quality^†^	Score ranges 0–21; higher score indicates worse sleep quality	65	4.0 (3.0, 7.0)	4.9 ± 0.4	64	4.0 (3.0, 7.0)	5.0 ± 0.5	68	5.0 (3.0, 6.0)	4.9 ± 0.4	0.57^‡^	0.94^‡^
Benefits and barriers of CGM and share												
Benefits	Score ranges 1–5; higher score indicates more of a benefit	69	4.0 (3.8, 4.4)	4.0 ± 0.1	69	4.6 (4.0, 5.0)	4.4 ± 0.1	69	4.8 (4.3, 5.0)	4.5 ± 0.1	<0.001^‡^	0.23^‡^
Barriers^b^	Score ranges 1–5; higher score indicates more of a burden	69	2.0 (1.9, 2.4)	2.1 ± 0.1	69	1.6 (1.1, 2.0)	1.7 ± 0.1	69	1.5 (1.3, 2.0)	1.6 ± 0.1	<0.001^‡^	0.60^‡^

^*∗*^Only participants who completed and had data available at the 52-week visit are included. ^†^Higher score indicates a worse outcome in questionnaire. For all others, a higher score is better. ^‡^*P*-value is from a signed-rank test. *P*-values are adjusted for multiple comparisons to control the FDR. ^§^*P*-value is from a paired *t*-test. *P*-values are adjusted for multiple comparisons to control the false discovery rate (FDR).

**Table 4:** Person-reported outcomes for the BGM–CGM group.

	BGM–CGM ^*∗*^										
		RCT baseline			26 Weeks (observational extension baseline)			52 Weeks			*P*-value 26 vs. 52 weeks
	Description	*N*	Median (Q1, Q2)	Mean ± SD	*N*	Median (Q1, Q2)	Mean ± SD	*N*	Median (Q1, Q2)	Mean ± SD	
PAID^†^	Score ranges 0–100; higher score indicates more of a problem	70	23.5 (10.0, 40.0)	27.0 ± 2.4	70	20.0 (7.0, 37.0)	25.2 ± 2.5	70	21.0 (8.0, 34.0)	22.8 ± 2.4	0.08^‡^
Glucose monitoring satisfaction	Score ranges 1–5; higher score indicates more satisfaction	70	3.6 (3.3, 3.9)	3.6 ± 0.1	70	3.7 (3.1, 3.9)	3.6 ± 0.1	70	3.9 (3.6, 4.3)	3.9 ± 0.1	0.002^§^
Hypoglycemia confidence^†^	Score ranges 1–4; higher score indicates more confidence	70	3.5 (3.0, 4.0)	3.4 ± 0.1	70	3.7 (3.1, 4.0)	3.6 ± 0.1	70	3.9 (3.1, 4.0)	3.6 ± 0.1	0.32^‡^
Diabetes technology attitudes	Score ranges from 6 to 30; higher score indicatefs a more positive attitude toward diabetes technology	70	23.0 (20.0, 25.0)	22.0 ± 0.4	70	23.0 (20.0, 25.0)	21.7 ± 0.6	70	25.0 (21.0, 25.0)	23.5 ± 0.3	0.002^‡^
Sleep quality^†^	Score ranges 0–21; higher score indicates worse sleep quality	65	5.0 (3.0, 6.0)	4.6 ± 0.3	66	4.0 (3.0, 6.0)	4.5 ± 0.3	67	4.0 (3.0, 6.0)	4.6 ± 0.3	0.60^‡^
Benefits and barriers of CGM and share											
Benefits	Score ranges 1–5; higher score indicates more of a benefit	70	4.0 (3.6, 4.5)	4.0 ± 0.1	70	4.2 (3.9, 4.8)	4.2 ± 0.1	70	4.6 (4.1, 5.0)	4.5 ± 0.1	<0.001^‡^
Barriers^†^	Score ranges 1–5; higher score indicates more of a burden	70	2.1 (1.9, 2.6)	2.2 ± 0.1	70	2.0 (1.4, 2.4)	1.9 ± 0.1	70	1.8 (1.3, 2.1)	1.7 ± 0.1	<0.001^‡^

^*∗*^Only participants who completed and had data available at the 52-week visit are included. ^†^Higher score indicates a worse outcome in questionnaire. For all others, a higher score is better. ^‡^*P*-value is from a signed-ranktest. *P*-values are adjusted for multiple comparisons to control the FDR. ^§^*P*-value is from apaired *t*-test. *P*-values are adjusted for multiple comparisons to control the false discovery rate (FDR).

## Data Availability

Data underlying the results of this study can be found at https://public.jaeb.org/datasets/diabetes.

## Supplementary Materials

Supplementary Materials Table S1: participant characteristics at start of extension phase (26 weeks). Table S2: effect of SHARE use at 52 weeks.
