# Supplementary material for: Adolescent- and Young Adult-Reported Outcomes and Use of Continuous Glucose Monitoring Features: A Report from the CITY Trial
Source: Pediatr Diabetes. 2023 Sep 15;2023:6906023. doi: 10.1155/2023/6906023 (PMC12016964; doi:10.1155/2023/6906023)
Supplement: Supplementary Materials — Table S1: participant characteristics at start of extension phase (26 weeks). Table S2: effect of SHARE use at 52 weeks. [file 6906023.f1.docx]

**Online Supplemental Material**

**CITY Study Group**

A listing of the CGM Intervention in Teens and Young Adults with Type 1 Diabetes (T1D) (CITY) sites with participating principal investigators (PI), co-investigators (I), primary coordinator (PC) and coordinators (C) is included below:

**Joslin diabetes Center, Harvard University, Boston, MA** Lori Laffel, MD, MPH (PI); Dayna McGill, MD (I); Emily Freiner, MSN, RN, NP-C (PC); Alan Schultz, MSN, RN, CPNP (C); Hannah Desrochers, MSN, RN, CPNP (C); Nisha Naik, BA (C)

**University of Colorado/Denver, Barbara Davis Center for Diabetes Aurora, CO** Paul Wadwa, MD (PI); Laurel Messer, RN, MPH, CDE, PhD (I); Todd Alonso, MD (I); Shideh Majidi, MD (I); Emily Simmons, BA (PC); Isabel Weber, MS, RD (C); Michelle Clay, BSN (C); Alex Coakly, BS (C); Tyler Reznick-Lipina, BS (C)

**Stanford University, Stanford, CA** Priya Prahalad, MD, PhD (PI); Darrel Wilson, MD (I); Bruce Buckingham, MD (I); Ryan Kingman, BS (PC); Marissa Ann Town, RN, BSN, CDE (C);

**International Diabetes Center/Park Nicollet/HealthPartners Institute Minneapolis, MN** Amy Criego, MD, MS (PI); Shannon Beasley, NP (I); Sean Dunnigan, RN, BSN (PC); Kathleen McCann, RN, BA (C)

**Yale University School of Medicine, New Haven, CT** Jennifer Sherr, MD, PhD (PI); Kate Weyman, MSN, APRN, FNP-C, CDE (I); Eileen Tichy, MMSc, PA-C, RD, CDE (I); Katie Gibbons, MD (I); Amy Steffen, RN, BSN (PC); Jennifer Finnegan (C)

**Naomi Berrie Diabetes Center, Columbia University New York City, NY** Robin Goland, MD (PI); Kristen Williams, MD (I); Sarah Pollak, RN (PC); Eberechi Cecilia Uche, BA (C); Courtney Sahn, RD (C); Analia Alvarez, RN (C); Courtney Melrose, RDN (C); Elizabeth Robinson, BA (C)

**University of North Carolina Diabetes Care Center Chapel Hill , NC** Katherine Bergamo, BSN, MSN, FNP-C (PI); John Buse, MD, PhD (I); Jean Dostou, MD, FACE (I); Marian Sue Kirkman, MD (I); Laura Young, MD, PhD (I);  Alexander Kass, BSN, RN, CDE (PC); Julie Uehling, BA, MS, CCRP (C)

**SUNY Upstate Medical University Syracuse, NY** Ruth Weinstock, MD, PhD (PI); Angela Mojica, MD (I); Suzan Bzdick, RN, CDE (PC); Patricia Conboy (C)

**Rocky Mountain Diabetes & Osteoporosis Center, Idaho Fall, ID** David Liljenquist, MD (PI); Carl Vance, MD (I); Mark Sulik, PharmD, CCRP (I); William Hardee, BS, CRC (PC); Christine Duval, BS, CCRC (C)

**Children’s Hospital Los Angeles, Los Angeles, CA** Roshanak Monzavi, MD (PI); Jennifer Raymond, MD, MCR (I); Daniel Brimberry, PhD (PC); Debra Miller, RN, BSN, CDE (C); Daniel Bisno, BA (C)

**Vanderbilt University Medical Center, Nashville, TN** Jill Simmons, MD (PI); Jennifer Kelley, MD (I); George Williams, RN, CDE (PC)

**Children’s Hospital of Philadelphia, Philadelphia, PA** Steven Willi, MD (PI); Pantea Minnock, RN, MSN, CPNP (I); Diana Olivos, MS (PC); Fiona Stuart, BSN, RN (C); Brian Grant, BSN, RN, CDE (C); Jennifer Smith, MPH (C)

**Baylor College of Medicine, Houston, TX** Daniel J. DeSalvo, MD (PI); Sarah K. Lyons, MD (I); Mary-Kylie DeLaO, MSN, RN, CDE (PC)

**Children’s Mercy Hospital, Kansas City, MO** Mark Clements, MD, PhD (PI); Wayne Moore, MD, PhD (I); Ryan McDonough, DO (I); Sarah Tsai, MD, FRCPC (I); Terri Lutjen, MS, RN, CPNP, CCRC (I); Jennifer James, BS, CCRC (PC); Heather Harding, RN (C); Stephen Orlich, RN, ACRP-CP (C)

**Jaeb Center for Health Research Tampa, FL** Kellee M. Miller, PhD; Thomas Mouse, BS, Nicole Reese, BS, David McNabb, AS, Heidi Strayer, PhD, Kamille Janess, BS, Israel Mahr, MS, Lauren Kanapka, MSc, Craig Kollman PhD, Roy Beck, MD, PhD

**CITY Operations Committee Members:** Mark Clements, MD, PhD, Daniel DeSalvo, MD, Korey Hood, PhD, Lauren Kanapka, MSc, Lori Laffel, MD, MPH, Laurel Messer, PhD, RN, MPH, CDE, Kellee Miller, PhD, Thomas Mouse, BS, Jennifer Sherr, MD, PhD,

Ruth Weinstock, MD, PhD

**Data and Safety Monitoring Board (DSMB)** Diane Wherrett, MD, FRCPC, Randi Streisand, PhD, Leslie Plotnick, MD

# Supplemental Table 1. Participant Characteristics at Start of Extension Phase (26 weeks)

|  | **Overall^a^**  **(N=140)** | **CGM-CGM^a^**  **(N=70)** | **BGM-CGM^a^**  **(N=70)** |
| --- | --- | --- | --- |
| **Age (years)** |  |  |  |
| 14-<19 | 88 (63%) | 45 (64%) | 43 (61%) |
| 19-<25 | 52 (37%) | 25 (36%) | 27 (39%) |
| *Median (Q1, Q3)* | 17.8 (16.1, 20.6) | 17.8 (15.8, 20.5) | 17.9 (16.4, 20.7) |
| *Range* | 14.6 to 25.5 | 14.6 to 25.5 | 14.6 to 25.5 |
| **Diabetes Duration (years)** |  |  |  |
| *Median (Q1, Q3)* | 8.5 (5.8, 13.9) | 8.2 (5.7, 13.2) | 9.7 (6.1, 14.3) |
| *Range* | 1.7 to 21.7 | 1.7 to 21.3 | 1.9 to 21.7 |
| **Gender: Female^c^** – *N (%)* | 68 (49%) | 30 (43%) | 38 (54%) |
| **Race/ethnicity^c^** – *N (%)* |  |  |  |
| White non-Hispanic | 87 (63%) | 46 (67%) | 41 (59%) |
| Black non-Hispanic | 9 (6%) | 2 (3%) | 7 (10%) |
| Hispanic or Latino | 31 (22%) | 17 (25%) | 14 (20%) |
| Asian | 6 (4%) | 1 (1%) | 5 (7%) |
| American Indian/Alaskan Native | 1 (<1%) | 0 (0%) | 1 (1%) |
| More than one race | 5 (4%) | 3 (4%) | 2 (3%) |
| **Annual Household Income^c^** – *N (%)* |  |  |  |
| < $25,000 | 10 (9%) | 6 (11%) | 4 (7%) |
| $25,000 - <$35,000 | 20 (18%) | 7 (13%) | 13 (22%) |
| $35,000 - <$50,000 | 17 (15%) | 4 (7%) | 13 (22%) |
| $50,000 - <$75,000 | 18 (16%) | 8 (15%) | 10 (17%) |
| $75,000 - <$100,000 | 16 (14%) | 11 (20%) | 5 (9%) |
| $100,000 - <$200,000 | 27 (24%) | 15 (27%) | 12 (21%) |
| ≥ $200,000 | 5 (4%) | 4 (7%) | 1 (2%) |
| **Health Insurance^c^** – *N (%)* |  |  |  |
| Private | 81 (58%) | 41 (59%) | 40 (57%) |
| Public | 58 (42%) | 28 (41%) | 30 (43%) |
| **Insulin Route** – N (%) |  |  |  |
| Injections | 64 (46%) | 36 (51%) | 28 (40%) |
| Pump | 76 (54%) | 34 (49%) | 42 (60%) |
| **HbA1c^d^ (%) [mmol/mol]** |  |  |  |
| *Mean ± SD* | 8.7 ± 1.2  [72 ± 13] | 8.5 ± 1.2  [69 ± 13] | 8.9 ± 1.2  [74 ± 13] |
| *Range* | 6.1 to 12.6  [43 to 114] | 6.1 to 12.0  [43 to 108] | 6.6 to 12.6  [49 to 114] |

^a^140 participants who completed extension for the RCT Treatment groups are included in this table.

^b^Missing data: Race/ethnicity 1 (<1%), annual household income 27 (19%), health insurance 1 (<1%)

^c^Only assessed at screening.

^d^26 week central lab value where available, otherwise the local screening value.

# Supplemental Table 2. Effect of SHARE Use at 52 Weeks

|  | **SHARE application users^a^**  **N=69** | **SHARE non-application users^a^**  **N=34** | **P-value SHARE vs. not using SHARE^b^** |
| --- | --- | --- | --- |
| GMSS- *Median (Q1, Q3)* | 4.1 (3.6, 4.3) | 4.1 (3.7, 4.6) | 0.78 |
| Benefits of CGM- *Median (Q1, Q3)* | 4.8 (4.3, 5.0) | 4.8 (4.4, 5.0) | 0.78 |
| Burden of CGM- *Median (Q1, Q3)* | 1.5 (1.1, 2.0) | 1.6 (1.4, 2.0) | 0.48 |
| CGM hours- *Median (Q1, Q3)* | 600 (426, 651) | 580 (389, 619) | 0.34 |

^a^Only participants who completed and had data available at the 52 week visit, had non-zero, non-missing CGM use, and were using the mobile application are included.

^b^P-values are a linear regression model with adjustment for treatment group. P-values are adjusted for multiple comparisons to control the false discovery rate (FDR).
